# Hepatic Abscess

**Published:** 1854-11

**Authors:** F. R. Payne

**Affiliations:** Marshal, Ill.


### ART. II—*Hepatic Abcess.* By Dr. F. R. Payne, Marshal, Ill.

The liver is liable to various forms of disease, both in this country and in Europe. But notwithstanding the multiform diseases to which this organ is exposed, and the frequency of their occurrence in every climate, all intelligent practitioners are aware that their discrimination is attended with much difficulty; consequently reliable judges in their treatment are but few. The great precision which recent developments of science has given to our knowledge of many other diseases, is well calculated to stimulate medical men to a more thorough investigation of those which are but imperfectly understood. *Suppurative inflammation* of this organ is not of frequent occurrence in this country. During a practice of eleven years, in the Wabash Valley, I have met with but one case, and the fortunate terminaticn of the disease uuder the most unfavorable circumstances, together with the unusual amount of matter discharged, has induced me to hope that a brief report will not be uninteresting to the members of this society.

Oct. 18th.—Mrs. M., aged thirty-six years, robust constitution, and mother of seven children. She had been under the charge of another physician for about fifteen days previous to my seeing her, and it seems the Doctor was of the opinion that she was suffering from the effects of a disease which he denominated, "*Fall Fever.*" From the imperfect history of the case, up to this time, which was derived from the patient and friends, it was difficult for me to determine what had been the early character of the disease. But I was induced to believe it had been *bilions remittent fever*, but had now assumed a typhoid type, with much hepatic disturbance. Her pulse was 120, tongue dry, bowels costive, pain in the right hypochondrium. Ordered three alterative portions sub. mur. hyd. to be carried off in eight hours after last powder, with Olei Ricinum and Terebinth. Dover powders after medicine operates and blister over region of the liver.

19th. Not much improvement. Continued treatment.

20th. Found her very restless, pain extends to the right shoulder and down the side. She is very much debilitated, bowels costive. Ordered wine and laxatives.

22d. Hepatic disturbance increasing, liver enlarged. Pulse 110. A remission of fever during the morning. Gave cathartic dose Pilula Hydg. and Quinine, Morphine and sup. Carb. Ferri, during the remission.

24th. Patient better; tongue moist; bowels have been freely moved; has some desire for nourishment. She was visited frequently by Dr. H. R. Payne and myself up to the 12th of Nov. At that time she had pain and tension on the right side, great emaciation, bowels costive, shivering and chilly sensations, tumor large, extends to hypogastric region. Ordered nitro-muriatic acid bath and continued a supporting treatment. At this time I was fully satisfied that my former suspicions were correct, and that the previous inflammation of the liver had terminated in suppuration. I

explained the character of the malady to the patient and her friends, and urged the propriety of an operation as early as practicable.

Nov. 27th, which was about eight weeks from the time the first symptoms made their appearance, Dr. C. L. Duncan and I visited her and found a slight elevation of the cuticle, with œdema and tenderness about 1½ inches from spinous process of the last dorsal vertebra on the right side, into which the lancet was introduced and followed by a copious discharge of pus. The treatment ordered at this time was tonics and stimulants, with an occasional laxative, as the bowels would not perform their function without artificial interference. The pus continued to flow for three weeks, during which time she used iodine internally and externally, in connexion with tonics and stimulants. I would not hesitate to fix the amount of pus discharged at two gallons, but we have no means of ascertaining the exact amount. It has not been my object to give the minutes of treatment in the above case, as there was nothing peculiar in it. The fortunate termination of the disease under such unfavorable circumstances, must be attributed, in a great measure to the *vis medacatrix nature*, or conservative power of the system. Was it not for this power of the system, every intelligent physician is aware that a deep-seated abscess in a vital organ would necessarily prove fatal. The patient now enjoys good health.

